# Inline myocardial t2* mapping with iterative robust fitting

**DOI:** 10.1186/1532-429X-13-S1-P308

**Published:** 2011-02-02

**Authors:** Saurabh Shah, Hui Xue, Andreas Greiser, Peter Weale, Taigang He, David N Firmin, Dudley J Pennell, Sven Zuehlsdorff, Jens Guehring

**Affiliations:** 1 Siemens Healthcare, Chicago, IL, USA; 2 Siemens Corporate Research, Princeton, NJ, USA; 3 Siemens AG, Erlangen, Germany; 4 Royal Brompton Hospital, London, UK

## Introduction

Myocardial T2* measurement is a valuable tool for non-invasive assessment of iron overload, and is clinically employed for planning and monitoring iron-chelating treatments for transfused thalassemia major patients [1]. Presently, for T2* assessment, dark-blood prepared gradient echo (GRE) images are acquired at multiple echo times (TEs). Thereafter, these images are analyzed within offline software such as CMRTools:ThalassaemiaTools®, in which the septal signal of a full thickness ROI is fitted to a monoexponential decay curve to estimate myocardial T2* [2]. The goal of this study was to develop and test a T2* measurement technique with automated inline T2*-map generation. Availability of such a technique on commercial MR systems may further utilization of such measurements in this patient group.

## Methods

An ECG-triggered 2D multi-echo GRE sequence was implemented on a 1.5T MR scanner (MAGNETOM Espree, Siemens AG) with support for dark-blood preparation. To generate an inline T2*-map, an integrated image reconstruction performs pixel-wise T2* estimation using a robust fit, in which the signal at each TE is iteratively weighted to reflect its fidelity to monoexponential decay curve. Points farther from the ideal relaxation curve are weighted lower, reducing their influence on the fit.

In five healthy volunteers, the method was used to acquire short axis images of the heart, accompanied by inline T2*-map computation. Additionally, to compare the accuracy of the robust-fit with a validated method, T2*-maps were retrospectively computed using multi-echo images of 32 patients. In all cases, a septal region-of-interest was manually drawn to obtain an average T2* value.

## Results

**Figure 1:**
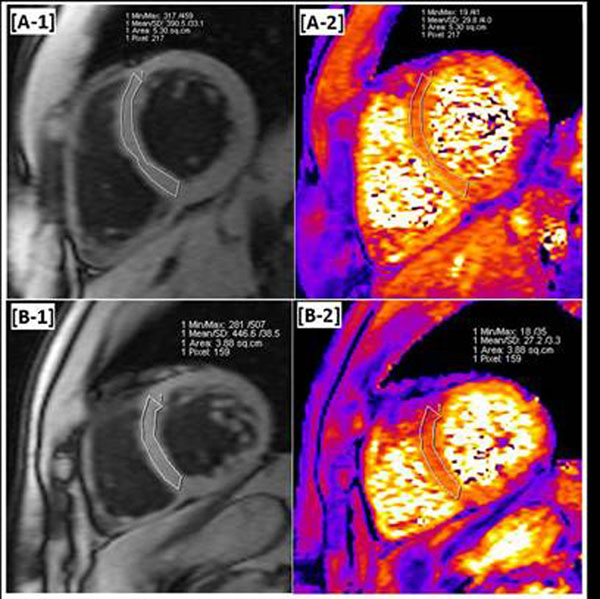
DB-prep GRE images [A-1, B-1] and corresponding T2*-map [A-2, B-2] produced using inline analysis in two healthy volunteers. The contours of these images mark septal regions from which the average T2* value was estimated. The average T2* value within septal regions were 29.8 ± 4.0 ms and 27.2 ± 3.3 ms for these two subjects, which are significantly above T2* < 20ms range indicating cardiac iron overload.

**Figure 2:**
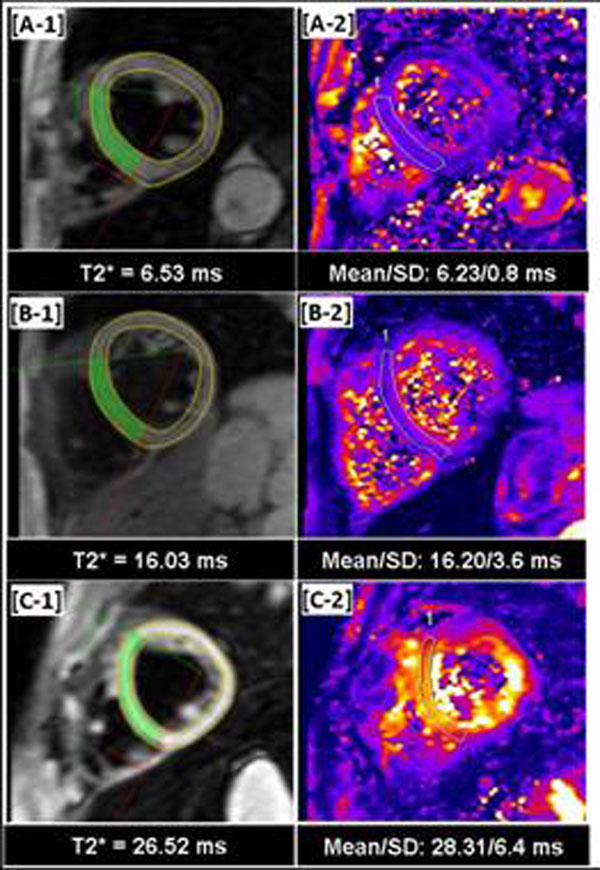
T2* estimates in 3 patients with suspected iron overload. [A-1, B-1, C-1] A DB-prep GRE image showing the region used to T2* estimate within CMRTools. The estimated T2* is listed directly below each image. [A-2, B-2, C-2] Corresponding T2* maps obtained with inline analysis. Average of pixel-wise T2* estimate was obtained from indicated septal region. In all 3 cases, the average value obtained from T2*-map closely matches the one calculated using CMRTools.

**Figure 3:**
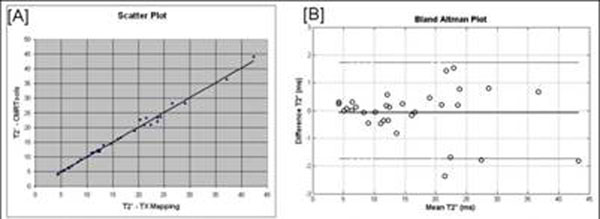
Statistical comparison between CMRTools and inline T2*-maps. 32 patients with suspected iron overload were retrospectively analyzed using both methods. The classification of each patient with severe (T2* <10ms; 10 patients), mild or moderate (10ms < T2* < 20ms; 11 patients) or no iron overload (T2* > 20ms; 11 patients) group was identical for both methods. [A] shows scatter plot compating T2* values estimated using CMRTools to those obtained using inline T2*-maps. Linear Regression: slope=1.01, intercept=-0.12 and R^2^-0.996. [B] is a Bland-Altman plot compating T2* estimates using two methods within these patients. Dotted lines indicate 95% confidence intervals.

Fig. 1 shows the T2*-maps obtained in two volunteers. Fig. 2 demonstrates T2* estimates using the inline T2*-maps in 3 patients with suspected iron overload, which match closely with the values obtained using CMRTools. Fig. 3 illustrates statistical comparison of T2* estimates using CMRTools and inline analysis in all 32 patients.

## Conclusions

The proposed technique computes pixel-wise T2* estimate which differs from region-based T2* assessment within CMRTools; however, the average T2* values within septum are highly correlated (R2=0.996) with the region-based estimates obtained using CMRTools. This is an encouraging result given that T2*-map is generated on the scanner without any need for user intervention to eliminate outliers, and that assessment of myocardial T2* is possible immediately following data acquisition. Prospective clinical studies are warranted to thoroughly validate this proposed method.
